# Toxicological Profile of Nanostructured Bone Substitute Based on Hydroxyapatite and Poly(lactide-co-glycolide) after Subchronic Oral Exposure of Rats

**DOI:** 10.3390/nano10050918

**Published:** 2020-05-09

**Authors:** Smiljana Paraš, Dijana Trišić, Olivera Mitrović Ajtić, Bogomir Prokić, Damjana Drobne, Slavoljub Živković, Vukoman Jokanović

**Affiliations:** 1 Faculty of Science and Mathematics, University of Banja Luka, 78000 Banja Luka, Republic of Srpska, Bosnia and Herzegovina; smiljana.paras@pmf.unibl.org; 2 Faculty of Dental Medicine, University of Belgrade, 11000 Belgrade, Serbia; dijana.trisic@stomf.bg.ac.rs (D.T.); s.zivkovic@stomf.bg.ac.rs (S.Ž.); 3 Department for Molecular Oncology, Institute for Medical Research, University of Belgrade, 11000 Belgrade, Serbia; oliveram@imi.bg.ac.rs; 4 School of Veterinary Medicine, University of Belgrade, 11000 Belgrade, Serbia; bogomirprokic@vet.bg.ac.rs; 5 Biotechnical Faculty, University of Ljubljana, 1000 Ljubljana, Slovenia; Damjana.Drobne@bf.uni-lj.si; 6 Department of Atomics Physics, Vinča Institute of Nuclear Sciences, University of Belgrade, 11000 Belgrade, Serbia; 7 ALBOS d.o.o., 11000 Belgrade, Serbia

**Keywords:** hydroxyapatite, bone substitute, genotoxicity, subchronic toxicity, biocompatibility

## Abstract

Novel three-dimensional (3D) nanohydroxyapatite-PLGA scaffolds with high porosity was developed to better mimic mineral component and microstructure of natural bone. To perform a final assessment of this nanomaterial as a potential bone substitute, its toxicological profile was particularly investigated. Therefore, we performed a comet assay on human monocytes for in vitro genotoxicity investigation, and the systemic subchronic toxicity investigation on rats being per oral feed with exactly administrated extract quantities of the nano calcium hydroxyapatite covered with tiny layers of PLGA (ALBO-OS) for 120 days. Histological and stereological parameters of the liver, kidney, and spleen tissue were analyzed. Comet assay revealed low genotoxic potential, while histological analysis and stereological investigation revealed no significant changes in exposed animals when compared to controls, although the volume density of blood sinusoids and connective tissue, as well as numerical density and number of mitosis were slightly increased. Additionally, despite the significantly increased average number of the Ki67 and slightly increased number of CD68 positive cells in the presence of ALBO-OS, immunoreactive cells proliferation was almost neglected. Blood analyses showed that all of the blood parameters in rats fed with extract nanomaterial are comparable with corresponding parameters of no feed rats, taken as blind probe. This study contributes to the toxicological profiling of ALBO-OS scaffold for potential future application in bone tissue engineering.

## 1. Introduction

Increased number of skeletal system disorders, due to the increase of the human lifespan, imposes a need for a new and more efficient bone healing procedure, since conventional treatments with auto- and allografts have many drawbacks, such as infections, pain, long-term recovery, and rejection by a host immune system [1,2]. Tissue engineering, as an interdisciplinary approach integrating biology, medicine, and engineering, offers an alternative by developing synthetic bone substitutes, which are able to provide bone repair and regeneration, even at the large scale bone defects [1,3,4].

Biocompatible porous three-dimensional (3D) scaffolds that mimic the natural bone structure have given the most promising results due to their biostimulative effect on cells proliferation and neovascularization [1,2,3,4,5]. Besides, these scaffolds have to mimic bone in all biological aspects and disintegrate at a rate that is similar to the new bone production to achieve the most efficient healing treatment [6,7]. Among the wide range of biomaterials used for the synthesis of bone scaffolds [8], hydroxyapatite [HAP, Ca_10_(PO_4_)_6_(OH)_2_] is one of the most widely utilized, since it possesses structural and chemical similarity to natural bone. It is biocompatible, bioresorbable, and bioactive, and it enables excellent cell adhesiveness [4,9,10,11].

The main drawbacks of HAP represent its lower rate of degradation in comparison to natural bone, and its mechanical strength, which mostly depends on the microstructure. To overcome it, HAP was modified due to available nanotechnology and widely combined with synthetic polymers. These composite scaffolds exhibited better mechanical properties and bioactivity, in comparison to ceramics or polymer alone [9,10,12]. Bearing all that in mind, novel porous bone substitute that is based on nanostructured HAP covered with thin polymeric film of poly(lactide-co-glycolide) (PLGA) has been synthesized recently and showed to present excellent biocompatibility through many in vitro and in vivo investigations, as well as numerous superior characteristics in comparison to commercially available product [13,14,15]. 

However, after being implanted in the tissue, the degradation process of the material in local tissue occurs, and dissolved products obtain to systematic circulation. The chronical presence of scaffolds’ products in circulation could lead to toxicity, and this aspect of nanostructured HAP has been poorly investigated so far. 

Systemic subchronic toxicity studies, usually conducted in rodents by repeated administration of tested compounds for more than 90 days (defined by the international standard ISO 10993: Biological evaluation of medical devices), are necessary before clinical application of bone substitute, especially at the large scale bone defects. A combined investigation of genotoxicity in vitro and systemic chronic toxicity could provide new insights in safety evaluation and potential long-term adverse effects of investigated materials and its leachable products [16].

The aim of this study was to provide toxicological profile based on in vitro genotoxicity and in vivo systemic subchronic toxicity. A range of endpoints was evaluated, including biochemical analyses, and histological and stereological measurements on liver, kidney, and spleen tissues, after 120 days of oral administration. This study contributes valuable understanding of the novel three-dimensional (3D) nanohydroxyapatite-PLGA scaffold (ALBO-OS) interaction on the systemic level to organism under subchronic exposure scenario. 

## 2. Materials and Methods 

### 2.1. Test Material

A novel composite scaffold that was based on calcium hydroxyapatite and poly(lactide-co-glycolide) (PLGA), named ALBO-OS, was used for in vivo toxicity study. The detailed procedure of material’s synthesis and characterization is explained in our previous investigations [13,14,15], and a summary is given in this paper. 

#### Synthesis and Characterization

Briefly, HAP powder was hydrothermally synthesized from an approximately stoichiometric mixture of calcium hydroxide (Ca(OH)_2_) and (NH_4_)_2_HPO_4_ (p.a. Merck KGaA, Darmstadt, Germany). The obtained hydroxyapatite powder was further used for the production of HAP granules. Further, ceramic slurry was made by mixing HAP granules and starch with water, and then poured over polyurethane foam with the required pore size distribution and dried at room temperature. Finally, it was heated in an oven at 600 °C and sintered at 1200 °C for 4 h. The obtained porous HAP compact was further disintegrated into 300 μm–1 mm granules. Finally, PLGA (Durect Corporation, Birmingham, AL, USA, 50:50, *M* = 45,000–70,000) thin film was deposited onto the surface of HA granules to obtain final product.

### 2.2. Genotoxicity Investigations In Vitro

#### 2.2.1. Cell Exposure and Viability Evaluation

THP-1 cells were seeded in 12-well plates in concentration 15 × 10^4^ cells per well. The next day cells were exposed to ALBO-OS extract. Extract contained maximal concentrations of Ca^2+^ ions, which exact value, was previously exactly ordered by ICP. The concentration corresponded to concentration of released Ca^2+^ ions in saturated solution obtained after immersion of 0.05, 5, 10, and 50 mg/mL ALBO OS in 10 mL distilled water with previously adjusted pH at 7.37, during 120 h. For negative control, the cells were not treated with material, while cells exposed to methyl methanesulfonate (MMS) solution (40 µM) were used as a positive control. The cells were exposed to various treatments for 1 h, after which they were centrifuged (200× *g*, 5 min.) and resuspended in Dulbecco’s phosphate-buffered saline (DPBS, Sigma-Aldrich, Saint Louis, MO, USA). Prior to alkaline comet assay, Trypan blue exclusion assay [17] was used for cell viability test. Cells’ viability evaluation was performed by microscopic observation (AXIO Vert.A1; Carl Zeiss, Jena, Germany) at 400× magnification (with minimum 200 cells/sample). 

#### 2.2.2. Alkaline Comet Assay 

The comet assay (single cell gel electrophoresis assay) was used to detect possible single- strand DNA breaks, double-strand DNA breaks, alkali labile site, and incomplete excision repair sites [18]. The microscopic slides were pre-coated with 0.5% normal-melting point (NMP) agarose. Cells were mixed with 1% low-melting point (LMP) agarose and then applied onto slides. After agarose solidification, the additional layer of 1% LMP agarose was applied, and followed by the slides immersion for 60 min. in alkaline lysis solution (1 M NaCl, 0.1 M EDTA, 10 mM Tris-HCl, 0.1% N-lauroylsarcosine, 1% Triton X-100, 30 mM NaOH). The slides were further gently washed in distilled water, and immersed for 20 min. in cold electrophoresis buffer (0.3 M NaOH, 1 mM EDTA, pH > 13), followed by the electrophoresis (30 min. at 0.7 V/cm) in the same buffer. At the end of electrophoresis, the slides were neutralized with 0.4 M Tris-HCl (pH 7.5) and stained with ethidium bromide (10 µg/mL). An epifluorescent microscope (Axio Imager.Z1; Carl Zeiss, Jena, Germany) at 400× magnification was used for slides analysis. CometAssay software (developed by Prof. Igor Kononenko research group, Laboratory for Cognitive Modeling, Faculty of Computer and Information Science, University of Ljubljana) was used to analyze 50 randomly selected comets per slide. As the DNA damage parameter, the percentage of DNA presence in the comet’s tail (tail DNA (%)) was used. The measurements were done in three independent repeats. 

### 2.3. Systemic Subchronic Toxicity Investigation

The Ethical Committee of the Faculty of Veterinary Medicine, University of Belgrade and Ministry of Agriculture and Environment Protection of the Republic of Serbia approved the experimental protocol (decision number 01-831/2), and realized in accordance with recommendations of European Good Laboratory Practice (86/609 EEC) related to protection and use of laboratory animals.

#### 2.3.1. Experimental Protocol

The investigation included 21 Wistar rats (*Ratus norvegicus*), at the age 8–12 weeks, and body weight 240–260 g. Animals were placed in individual cages. During the experiment, the animals were kept under following conditions: temperature 23 ± 3 °C, air humidity 55 ± 5%, 8–12 air changes/h, and artificial lighting at intervals of 12 h day/night. The animals had free access to food and water. The animal health status was monitored daily during the experiment. 

Extracts that were obtained after the highest concentration of ALBO OS, obtained during 5 days immersion of material (100 mg/mL) in distilled water (in accordance with the standard ISO 10993-12:2012—Biological evaluation of medical devices—Part 12: Sample preparation and reference materials) was used for per oral rats feeding. After seven days of adaptation period, the animals were randomly divided into experimental (n = 12) and control group (n = 9). To the animals in the experimental group, 1 mL of ALBO-OS extract was per oral fed for 120 days, every day at the same time, while, for the control group, distilled water was used. During the experiment, control of the health status, behavior, changes in skin and haircut, food and water consummation, urination, and defecation, was performed and recorded daily. The body weight of animals was checked after the adaptation period, and afterward three times through the experiment.

#### 2.3.2. Blood Analysis

At the end of the experiment, the blood samples were taken from lateral tail vein for hematological (leukocytes, thrombocytes, hemoglobin) and biochemical analyses (alanine aminotransaminase (ALT), aspartate aminotransaminase (AST), urea, creatinine, bilirubin, serum protein, glucose, and alkaline phosphatase (ALP)).

### 2.4. Histological and Stereological Analysis 

The animals were sacrificed by intravenous administration of thiopentone sodium solution (170 mg/kg). The rats’ livers, kidneys, and spleens were removed, immersed in Buoin’s fixative solution for 24 h, and, for better absorption, further cut into smaller pieces and then immersed into fresh Buoin’s solution. Subsequently, all of the dissected organs were prepared for the light microscopy using the standard tissue preparation procedure. Tissue was embedded in paraffin. Tissue sections of 5 µm thickness were cut using rotary microtome (Leica rotary Microtome RM 2165, Leica Microsystems, Wetzlar, Germany). Every fifth section was used for the analysis in order not to repeat the analyzed structures. Tissue was stained with hematoxylin-eosin (H&E) (Merck KGaA, Darmstadt, Germany). The qualitative analysis of the microscopic slides was performed while using the light microscope (Leica DM8000 M with MEGA VIEW camera and software system for digital image transfer, Wetzlar, Germany). For quantitative, stereological analysis, micrographs were acquired in the RGB layout and converted to binary format. Measurements were made using a stereo-universal test system according to Cavalier’s principle, while using 16.0 point-counting system (MBF software system Application Suite 3.0.0, MBF Bioscience, Williston, VT, USA), with P2 spacing grid at the maximum 400× magnification. 

Stained tissue sections of liver, kidney, and spleen were used to measure the number and volume density (Vv) of epithelial cells. Volume density was calculated through the following formula: Vv = Pf/Pt (mm^0^), where Pf represents the number of the desired phase on the test system (epithelial cells) and Pt represents the total number of points of the test system [19].

The following principle was used for the determination of the number of matches on the test system: all of the endothelial cells’ nuclei were marked as reference point, while the cells surfaces were calculated according to the principles: (i) all cells that have a clearly visible contour and nucleus, (ii) the cells contour do not touch the test system frame, and (iii) the cells contour is clearly visible without a visible nucleus. The numerical density (Nv) of all epithelial cells was calculated based on the cell count (Q) in the volume of analyzed tissues (Vo). The volume of analyzed tissue was evaluated as the product of the number of counted frames (ΣPi), the space of counted frame (a = 25,002), and the height factor (h) (the histological section), as presented in the following formula [20]:

(1) $$\mathrm{Nv}=\frac{Q}{Vo}=\frac{Q}{\sum_{i=1}^{n}Pi\times a\times h}\;({\mathrm{mm}}^{-3})$$

MBF software system was used to measure the surface and volume of epithelial cells and nuclei, by the thickness of their diameters. Measuring the surface of epithelial cells and nuclei enabled the measurement of the cells, cytoplasm, and nuclei volumes. The ratio between the cells’ nuclei and cytoplasm volumes was used to determine the nucleocytoplasmic ratio (NCO). The mitotic index was determined as the ratio between the number of cells in mitosis and the total number of cells in 10 visible optical fields per slide, with maximum 200× magnification.

### 2.5. Immunohistochemical Analysis

For the immunohistochemical analysis, the paraffin tissue sections of rat liver, spleen, and kidney were analyzed cut at 5 µm, and the slides were heated for 60 min. at 56 °C. Prior to the antigen retrieval step, the sections were deparaffinized and rehydrated through the series of xylenes and alcohols. To block endogenous peroxidase activity, tissue sections were treated with 3% H_2_O_2_ solution in PBS. Tor epitope retrieval, tissue sections were heated in microwave oven for 21 min., at 680 W, in 10 mmol/L citrate buffer, pH 6.0. The tissue sections were incubated with appropriate antibodies (against CD68 (RTU-CD68, Leica, LOT 6000698, Wetzlar, Germany) and Ki67 protein (Monoclonal Mouse Anti-human Ki67 Antigen, Clone Ki-S5, Sigma-Aldrich, Saint Louis, MO, USA (dilution 1:100)) over night, at +4 °C, in the humid chamber. Streptavidin-biotin technique was used for immunostaining (LSAB+/HRP Kit, Peroxidase Labeling, K0690, DAKO Cytomation, Glostrup, Denmark). Immunoreactivity complex was visualized with DAKO Liquid DAB+ Substrate/Chromogen System (Dako, CA, USA, Code No. K3468) and then counterstained with Mayer’s hematoxylin (Merck, KGaA, Darmstadt, Germany). As negative control served the tissue sections with omitted primary antibody. For positive control, the tissue sections that were known to express CD68 and Ki67 were used. CD68 and Ki67 positive cells in tissues were analyzed by a light microscope (Olympus AX70, Hamburg, Germany) with 40× magnification.

In all of analyzed sample of tissue, five hot spots were selected for image analysis in ImageJ (The National Institutes of Health, Bethesda, MD, USA). Ki67 and CD68 expressions were calculated by determining the positive Ki67/CD68 areas (brown-colored cells) in microscopic fields (40× magnification), based on the threshold [21]. Median values of CD68 and Ki67 immunostaining were calculated for each individual tissue sample and then used for further analysis.

### 2.6. Statistical Analyses

All of the values were presented as mean ± standard deviation. Group comparisons were performed while using parametric (two-way ANOVA, one-way ANOVA followed by Dunnett’s test and *t*-test) or nonparametric tests (Kruskal–Wallis and Mann–Whitney U-test), depending on data distribution, with significance set at *p* < 0.05. Statistical software SPSS 20.0 (IBM corp., Armonk, NY, USA) was used for data processing.

## 3. Results

### 3.1. Genotoxicity Results

**Figure 1:**
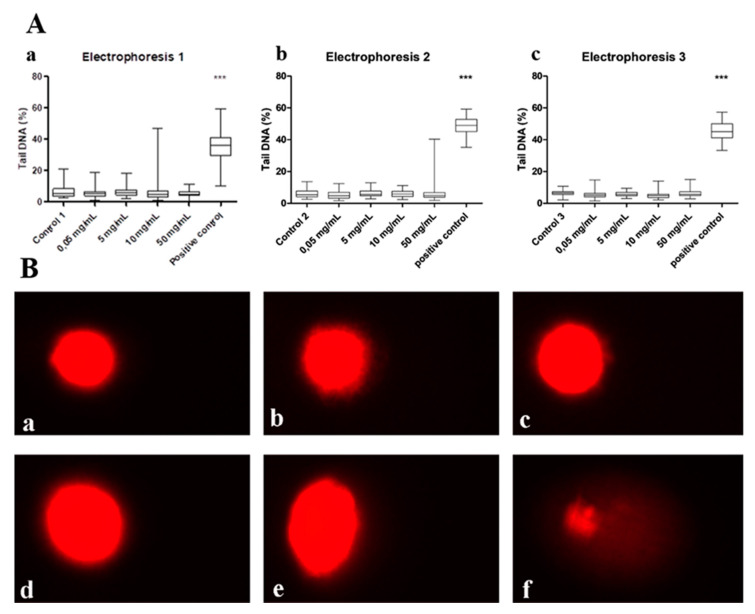
(**A**) Comet assay results: (**a**) first repeat, (**b**) second repeat, (**c**) third repeat. Asterisks denote the significant differences with respect to the untreated control cells (*** *p* < 0.001; one-way ANOVA, Dunnett’s test). (**B**) Images of comets for: (**a**) negative control, different concentration of material’s extract: (**b**) 0.05 mg/mL, (**c**) 5 mg/mL, (**d**) 10 mg/mL, (**e**) 50 mg/mL and (**f**) positive control.

The results of Trypan blue exclusion assay indicated that none of the nanoHAP-PLGA concentrations (0.05, 5, 10, and 50 mg/mL) was cytotoxic to THP-1 cells after 1 h exposure. In all tested samples, cell viability was over 90%. The results of the comet assay showed that none of the used ALBO-OS concentrations was genotoxic to THP-1 cells (Figure 1). 

There is no comet tail for any of material’s concentration, which means that there is no damaged DNK (broken fragments) that would migrate from the “head” during the electrophoresis, like in the case of positive control, as it can be seen in Figure 1B. 

### 3.2. Systemic Subchronic Toxicity Results

#### 3.2.1. Main Clinical Symptoms and Observations

**Figure 2:**
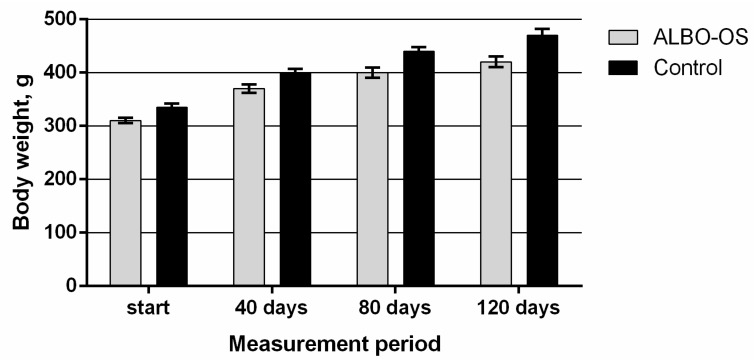
Average body weights of experimental and control animals during the study of chronic systemic toxicity of ALBO-OS.

During the course of the exposure, no adverse effects that were related to the behavior of the tested animals were observed, as investigated at a daily level. No changes in skin and haircut, as well as changes in food and water consummation, or urinating and defecation, have been reported. Body mass was measured four times during the experiment, and in all animals it was mildly and consistently increased through the observation period (Figure 2). 

#### 3.2.2. Results of Blood Analysis

The hemoglobin and thrombocytes values were fairly similar in the experimental and control group. Significantly higher leukocyte values were found in the control group, *p* < 0.001 (Table 1). 

In Table 2, the results of the analysis of the biochemical parameters are presented. No significant differences were found for urea, creatinine, and bilirubin values. The ALT level and glucose were slightly higher in the experimental group when compared to the control, while the AST level was higher in the control versus the experimental group, all without significant differences. The alkaline phosphatase values were higher in the experimental group, and this difference was highly statistically significant (*p* < 0.003).

#### 3.2.3. Histological and Stereological Analysis of the Liver Tissue

**Figure 3:**
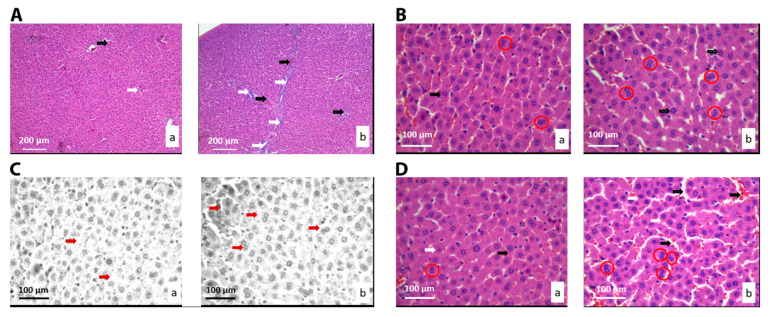
Micrographs of the histological cross-section of the liver of the control group (**a**) and treated group (**b**), (hematoxylin-eosin (H&E)). (**A**) White arrows show connective tissue and black show blood vessels. Magnification 20×; (**B**) Black arrows show hepatocytes’ nuclei, and red circles show hepatocytes with two nuclei. Magnification 50×; (**C**) Red scalpers show hepatocytes’ nuclei. Magnification 50×, digitally processed RGB technique; (**D**) Black arrows show capillary sinusoids, and red circles show hepatocytes with two nuclei. Magnification 50×.

The morphology of the liver tissue (of animals orally exposed to ALBO-OS extract) was not significantly different to the control. Hepatocytes and hepatic plaques were normal and neatly placed, without abnormality in the form of a central vein. There were no pathological changes in liver tissue, such as cysts, fibrosis, loss of hepatocytes, necrosis, or lymphocyte aggregates, due to inflammatory reactions, in the experimental or in control group. Further histological and cytological analysis of liver tissue was based on the comparison of fifteen stereological parameters (Table 3). The number and numerical density (*p* < 0.05), and surface area of capillary endothelial cells (*p* < 0.05) increased in the experimental group relative to control (Table 3 and Figure 3A). 

The digital processing of the cross-section of the liver tissue in the RGB program revealed an increase in the number, numerical density, and number of mitosis in treated group (Figure 3C). The hepatocytes with two nuclei were revealed in experimental group (Figure 3B,D, red circles), evidencing the higher number of mitosis, in comparison to the control. Although, the increase was not statistically significant (*p* > 0.05) (Table 3). 

#### 3.2.4. Histological and Stereological Analysis of the Kidney Tissue

**Figure 4:**
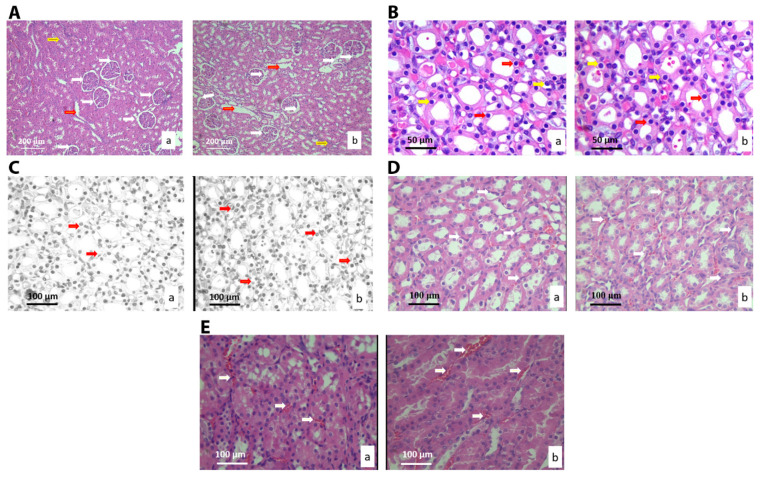
Micrographs of histological cross-sections of the kidney of the control (**a**) and treated group (**b**), (H&E). (**A**) White arrows show glomeruli, red arrows show blood sinusoids and yellow arrows show connective tissue. Magnification 20×; (**B**) Yellow arrows show nuclei, and red arrows show epithelial cells of collecting ducts. Magnification 50×; (**C**) Red arrows show the epithelial cells of collecting ducts. Magnification 50×; digitally processed RGB technique; (**D**) White arrows show the connective tissue of collecting ducts. Magnification 50×; and, (**E**) White arrows show the blood sinusoids of the collection ducts. Magnification 100×.

Histological analysis revealed no pathological changes in kidney tissue, such as the loss of cells of collecting ducts, loss of glomeruli, cysts, fibrosis, necrosis, or lymphocyte aggregates and nodules. Additionally, there was no infiltration of inflammatory cells, inflammation or edema in glomeruli. The volume densities of the epithelial cells of the collecting ducts, blood capillaries, connective tissue, and glomeruli were higher in the treated group when compared to the control (Table 4 and Figure 4A), with no statistical significance. The surface area of the epithelial cells, their nuclei, and the NCO ratio did not change significantly in the experimental group, as compared to the control (*p* > 0.05, Table 4, Figure 4B,C). Analysis regarding connective tissue and blood sinusoids showed their slight change in morphology (*p* > 0.05, Table 4, and Figure 4D). The surface area of glomeruli significantly decreased in the experimental group relative to the control (*p* < 0.05), while the surface of Bowman’s space slightly increased (Table 4 and Figure 4E).

#### 3.2.5. Histological and Stereological Analysis of the Spleen Tissue

**Figure 5:**
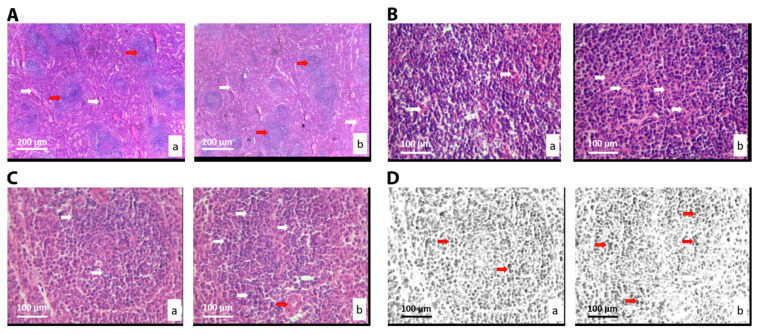
Micrographs of the histological cross-section of the rat spleen of the control (**a**) and treated group (**b**), (H&E). (**A**) White arrows show connective tissue and red arrows show lymphatic tissue. Magnification 200×; (**B**) White arrows show epithelial cells. Magnification 50×; (**C**) White arrows show lymphocytes and red arrows show blood vessel. Magnification 50× and, (**D**) Red arrows show lymphocytes. Magnification 50×; digitally processed RGB technique.

Histological analysis revealed no pathological changes in the spleen tissue, such as change in the morphology of the spleen tissue, loss of lymphocytes, cysts, fibrosis, loss of epithelial and radial cells, necrosis, or large lymphocytes accumulation. Epithelial, endothelial, and connective tissue cells have increased in their number, volume density, and numerical density in the treated group when compared to control (Table 5, Figure 5A,B), mostly with no statistical significance, except for the number of connective tissue cells (*p* < 0.05). 

The number and numerical density of lymphocytes in the experimental group were higher in comparison to control (*p* < 0.05, Table 5, Figure 5C,D). A large number of mature lymphocytes in sinusoids and between the star-shaped epithelial cells of the spleen were observed. Lymphocytes were highly colored, uniformly distributed, with no signs of focal aggregations in tissue. 

#### 3.2.6. Expression of Proteins Ki67 and CD68 in Liver, Kidney, and Spleen 

**Figure 6:**
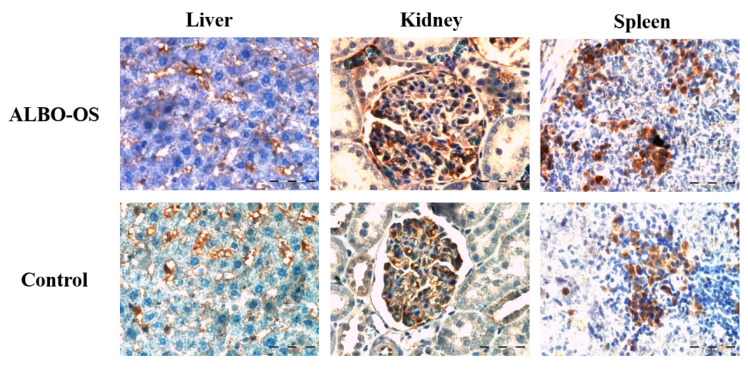
Representative images of Ki67 protein expression in rats’ liver, kidney and spleen tissue sections of experimental and control group, 40× magnification. Significant expression of Ki67+ cells was observed in all groups.

**Figure 7:**
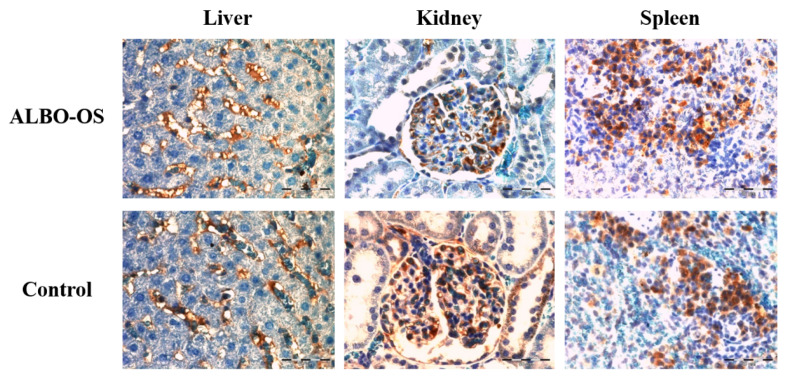
Representative images of CD68 protein expression in rats’ liver, kidney and spleen tissue sections of experimental and control group, 40× magnification. Mild expression of CD68+ immunoreactive cells was observed in all groups.

The highest average number of Ki67 positive cells was detected in kidney experimental group, while the smallest number of Ki67 positive cells were detected in liver control group, which indicated that the proliferation ratio was different in all three investigated organs (Figure 6). According to our results, the highest average number of immunoreactive CD68 cells was detected in spleen tissue samples of experimental group, while the smallest average number of cells were detected in the liver tissue samples (Figure 7). 

**Figure 8:**
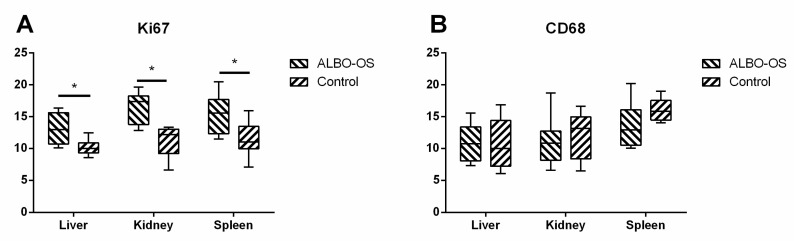
Percentage area of Ki67 (**A**) and immunoreactive CD68+ (**B**) stained cells was calculated with ImageJ using 3 images per organ. The results were analyzed with a two-way ANOVA, a *p* value < 0.05 was considered significant; * p < 0.05.

Differences between the average number of Ki67 positive cells was significantly higher in kidney tissue samples of experimental group in comparison to control (*p* < 0.05). Additionally, the average number of Ki67 positive cells was detected in liver (*p* < 0.05) and spleen (*p* < 0.05) experimental groups in comparison to control (Figure 8A). 

The differences between the number of immunoreactive CD68 cells was not statistically significant in all three groups of investigated organs (Figure 8B).

## 4. Discussion

This study aimed to characterize toxicological potential of ALBO OS scaffold by investigating its potential genotoxicity in vitro and systemic subchronic toxicity after oral exposure, which is very important for its potential application in dental medicine. 

Genotoxicity investigation on THP-1 cells using comet assay showed the lack of damaged DNAs in the cells that were cultivated in material’s extracts up to concentration 50 mg/mL which highly suggests that there were no changes inside of the cells on the level of DNA. This is in accordance with previous investigations that also reported low genotoxicity of hydroxyapatite and PLGA alone, which are the main components of the ALBO-OS [22,23,24].

The blood analysis showed that there were no significant changes in the hematological parameters between the experimental and control group. Neither myelotoxic nor autoimmune effects on peripheral blood cells were detected in the experimental groups. This is in agreement with previous reports on the low hazard of these material [13,25]. Among the analyzed biochemical parameters, only alkaline phosphatase was significantly higher in the experimental group, compared to the control. This difference indicated the potential of the absorption of calcium and phosphates from ALBO OS, which consequently might led to an increase of ALP serum [26]. 

The results of histopathological examination of systemic toxicity on liver, kidney and spleen sacrified rats after their oral exposure to extract of ALBO OS maximal concentration (100 mg/mL) during 120 days per oral feeding were particularly important for the determination of the toxicological profile ALBO OS. 

Accordingly, the liver is chosen due to its vital role in organism being reflected by its physiological functions, synthesis and detoxification of various metabolites. In other words, any lesions caused by drugs, chemicals, and pathological status could interrupt its functions [27]. The ALT level, which is found primarily in the liver, and is released into the circulation from damaged hepatocytes, was slightly higher in the experimental group, while the AST level, found in the erythrocytes, myocytes, and kidney cells also, was higher in the control, without significant differences [27]. Similar results of biocompatibility investigation of Mg-Zn materials were reported, with the assertion that there was no change in the hepatocyte architecture and morphology of liver lobules [28]. 

The increased mitotic index and the number of hepatocytes with two nuclei could be explained by the liver adaption to the presence of new material in the organism [29]. It could be assumed that, as a reaction to chronical presence of ALBO OS extract in organism, the physiological reaction of increased function occurred. It is interesting to note that immunostaining confirmed parenchymal proliferation over increased expression value of the Ki67, while a number of immunoreactive CD68 positive cells remained in the physiological range. The increase in Ki-67 protein expression might be caused by the fact that posttranscriptional regulatory mechanisms suppress protein synthesis until the cells are at the G1/S boundary playing a critical role in the regulation of mitosis. In this case, like too many similar evidences published in other research papers, it seems that Ki67 is involved in the regulation of cell cycle progression, including DNA replication, higher-order chromatin organization, etc. [30,31].

In our previous study, the effect of calcium silicate and calcium aluminate implantation on systematic subchronic toxicity was evaluated [32]. It was concluded that materials induced normal and reversible response in the liver. Namely, the proliferation of parenchymal cells in this case was also reported, with no visible signs of pathological changes or immunological reaction to the materials [32].

An increase in the number and density of capillary endothelial cells (*p* < 0.05) (obtained as all other histological and immunohistochemical parameters included in this paper, by observation 12 animals in experimental group and nine animals in the control group) indicated a statistically significant increased blood flow through the liver in experimental group, when comparing with control group of rats, which is characteristic for processes with more intense blood filtration. Additionally, significant increase the number of endothelial cells (*p* < 0.05) participating in the formation of new blood sinusoids was observed, which indicated that the process of the necessary adaptation of the rats’ liver to ALBO-OS is occurred. As it is well known, the liver sinusoidal endothelial cells (LSEC) constitute the sinusoidal wall, or endothelial lining. Therefore, they can be observed as unique capillaries that differ from other capillaries in the body, because of the presence of open pores or fenestrae lacking a diaphragm and a basal lamina underneath the endothelium. The increasing quantity of the Ca^2+^ ions, interacting with lipid soluble ionophores, which transport Ca^2+^ ions across liver cell membranes, can induce increased transport Ca^2+^ ions inside of the liver sinusoidal endothelial cells, influencing their contraction and, consequently blood flow diminishing through them. Therefore, the balance establishing in blood flow through sinusoids, the new blood sinusoids should be formed, simultaneously leading to the increased number of the endothelial cells [20,33,34,35]. The connective tissue cells, as stromal cells, did not change their structure or density, suggesting no pathological change on liver tissue occurred during four months of exposure. Similar to our results, in previous studies of systematic toxicity of new endodontic materials that are based on calcium hydroxyapatite and calcium silicates, an increased mitotic activity of parenchymal cells was observed and followed by the increased density of the cells and a moderate increase in the liver sinusoids and hepatocyte surface [36,37].

The serum levels of creatinine, as an indicator of kidney function and the change of renal histopathology [38], were very similar in the experimental and control group, as well as values of urea and bilirubin. In the previous study, the absence of any morphological changes in the kidney tissue due to the implantation of biocompatible Mg-Nd-Zn-Zr material into the bone of the rabbit was reported [39], which is in accordance with the presented results. 

The number of epithelial cells in collecting ducts and the connective tissue cells, as well as other stereological and cytological parameters, were not significant (*p* > 0.05), while immunostaining revealed significantly higher number of Ki67 positive cells, indicating cell proliferation in experimental group, increased kidney activity, and blood flow. In the study of mineral trioxide aggregate effect on the liver and kidney function, it was reported that the kidney function had subsequently increased after a month, while the increased liver function was observed during the first week and further increased during the observed period [29]. Glomeruli, the beneficial components of the kidneys influenced by all matter in the blood reacted by shrinkage in the presence of material’s products, while its morphology was preserved. Besides, the area of renal kidney glomeruli of rats (*p* < 0.05) was statistically significantly reduced during the treatment by ALBO-OS, probably as a consequence of the increase of filtration blood flow into the them. Fray has developed a mathematical model suggesting the stretch-dependent influx of extracellular calcium ions into juxtaglomerular cells to account for the pressure control of renin secretion [40]. In detail, an increase of the renal artery pressure would enhance the circumferential stretch of the juxtaglomerular cells, thereby causing membrane depolarization. 

Therefore, a rise of the intracellular calcium concentration inhibits reining secretion from these cells, as a consequence of the renal glomeruli shrinkage [29,41,42]. Additionally, the change was reflected in the slight increase in the surface of Bowman’s space as a sign of increased glomerular filtration. In our study, while the spleen retained a normal physiological architecture, the number of lymphocytes increased in the treated rats, thus indicating an immune response to ALBO OS extract. While proliferation of lymphocytes was induced by material during four months of oral administration, lymphocytes were highly colored, uniformly distributed, with no signs of focal aggregations in spleen tissue, suggesting mature lymphocytes were predominantly in the tissue, with no signs of tissue hyperplasia.

Furthermore, number of CD68 positive immune cells in spleen was similar in the experimental and control group. In previous investigations of systemic toxicity of new endodontic material based on hydroxyapatite and calcium silicates, the slight hypertrophic changes in white pulp were reported, with no adverse effect on the spleen function and its immune response activation [36]. Statistical analysis showed a significant increase (*p* < 0.05) of the number of connective cells in the spleen of rats, during the treatment with ALBO-OS, which is a consequence of the increased production of lymphocytes in the spleen and their increased activity in the blood. Additionally, the increase of the number and numerical density of lymphocytes in the spleen of rats during the treatment by ALBO OS was also statistically significant (*p* < 0.05). 

Calcium plays an essential role in the activation and maturation of lymphocytes. As a second messenger, calcium ions are involved in most biochemical processes that transduce the external signals into cell responses. An increase in Ca^2+^ ions and subsequent protein kinase C activation is indispensable for T-lymphocyte proliferation and an increase of their number [43]. Previous studies reported that increased Ca^2+^ ions promotes IL-2 mRNA gene expression and protein synthesis and that IL-2 is a potent T-lymphocyte growth factor [28,44,45]. Since spleen primarily acts as the main filter for blood-borne pathogens and antigens, an increased number of lymphocytes could be explained by higher blood perfusion through spleen and the presence of the materials’ extract at high concentration in organism during a prolonged time period [46]. Furthermore, haematological analysis revealed no increase of lymphocytes in systematic circulation in the experimental group, which implied the transient and physiological nature of increased spleen tissue lymphocytes. 

An estimate of blood volume in the rat is 4.7 to 8.0 mL per 100 g body volume (BW) (mean value 7.0 mL) [47]. The BW in our case for all rats were 240–260 g, from which follows that blood quantity following Lee and Blaufox research [47] was probably between 16.8–18.2 mL. Taking in mind that all of the experimental rats were fed with 1 mL/day of fresh saturated solution of extract obtained by immersion 100 mg/mL for 120 days, the total quantity of used extract for 120 days is 120 mL, which is 6.6–7.1 times higher than the total volume of the blood of rats. Although this is exceptionally high quantity of Ca^2+^ ions in rat’s blood, the pathological effect expressed over significant changes of morphology of tissue of the liver, kidney, and spleen were not registered, suggesting that the used nanomaterial is biocompatible. 

## 5. Conclusions

The tested ALBO OS proved that it could immitate natural bone by its arhitecture and mechanical properties, as well by the rate of resorption, as confirmed by our previous investigations of its structure and physico mechanical characteristics briefly described in the Appendix A of this paper. In additon, this material also showed low hazard potential, as determind by in vitro genotoxicity study and sistemic toxicity study. Genotoxic study confirmed no genotoxic effect, even at the highest concentration of the 50 mg ALBO OS/mL. Daily oral administration of ALBO OS extract extremely high concentration (100 mg/mL) through a prolonged time period (120 days of their per oral feeding) induced no adverse and reversible response in rats’ liver, kidney, and spleen. Despite the significant increase average number of the Ki67 suggesting cell proliferation, a slight increase in number of immunoreactive CD68 positive cells in the presence of ALBO OS, evidencing no difference in number of tissue immunoreactive cells, although the proliferation of parenchymal and stromal tissue occurred. This study suggests the good biocompatibility of novel nanostructured scaffold, which could serve as a suitable candidate for future investigations generally in regenerative medicine. 

## Figures and Tables

**Table 1:** Results of blood analysis; values are shown as mean ± SD (*** *p* < 0.001; *t*-test).

	ALBO-OS	Control
Leucocytes	4.12 ± 1.94	6.83 ± 0.77 ***
Hemoglobin	144 ± 8.26	140.8 ± 6.76
Thrombocytes	566.56 ± 269.54	713.20 ± 181.58

**Table 2:** Results of analysis of biochemical parameters; the values are shown as mean ± SD (*** *p* < 0.001; *t*-test).

	ALBO-OS	Control
ALT	74.50 ± 23.07	71.50 ± 31.11
AST	630.50 ± 637.22	844.32 ± 838.57
Urea	5.68 ± 0.81	5.21 ± 0.36
Creatinine	42.30 ± 5.50	40.00 ± 4.84
Bilirubin	1.12 ± 0.51	1.01 ± 0.32
Glucose	6.23 ± 1.70	5.48 ± 0.99
ALP	81.50 ± 13.91 ***	63.00 ± 9.80

**Table 3:** Stereological parameters of the liver of the control and treated groups of rats; values are shown as mean ± SD (* *p* < 0.05; *t*-test).

Parameter	ALBO-OS	Control
Volume density of hepatocytes (mm^0^)	0.681 ± 0.032	0.656 ± 0.045
Volume density of capillary sinusoids (mm^0^)	0.204 ± 0.008	0.163 ± 0.005
Volume density of connective tissue (mm^0^)	0.138 ± 0.004	0.126 ± 0.003
Number of hepatocytes	273,818.2 ± 24,146.0	255,168.4 ± 21,822.7
Numerical density of hepatocytes (mm^−3^)	47,887.4 ± 3119.6	44,575.3 ± 2632.9
Surface area of hepatocytes (μm^2^)	152.8 ± 4.1	148.5 ± 2.9
Surface area of hepatic nucleuses (μm^2^)	49.2 ± 1.9	46.8 ± 2.1
NCO of hepatocytes	0.383 ± 0.022	0.324 ± 0.023
Mitotic index of hepatocytes	1.77 ± 0.234	1.61 ± 0.24
Number of connective tissue cells	138,442.5 ± 16,443.4	127,486.1 ± 17,518.1
Numerical density of connective tissue cells (mm^−3^)	24,888.3 ± 3084.8	21,934.4 ± 2366.6
Surface area of connective tissue cells (μm^2^)	103.4 ± 2.4	99.6 ± 3.3
Number of capillary endothelial cells	328,132.2 ± 32,336.3 *	275,915.5 ± 29,079.0
Numerical density of capillary endothelial cells (mm^−3^)	53,714.3 ± 953.7 *	48,068.5 ± 2806.7
Surface area of capillary endothelial cells (μm^2^)	84.5 ± 4.6	75.1 ± 3.9

**Table 4:** Stereological parameters of kidneys of the control group and treated group of rats; values are shown as mean ± SD (* *p* < 0.05; *t*-test).

Parameter	ALBO-OS	Control
Volume density of collecting ductus’ epithelial cells (mm^0^)	0.34 ± 0.04	0.33 ± 0.04
Volume density of blood sinusoids (mm^0^)	0.26 ± 0.03	0.23 ± 0.03
Volume density of connective tissue (mm^0^)	0.11 ± 0.01	0.10 ± 0.01
Volume density of glomeruli (mm^0^)	0.32 ± 0.04	0.30 ± 0.04
Number of collecting ducts’ epithelial cells	139,562 ± 1523	138,147 ± 3332
Numerical density of collecting ducts’ epithelial cells (mm^−3^)	20,107 ± 4116	19,572 ± 2621
Surface area of collecting ducts’ epithelial cells (μm^2^)	209 ± 43	204 ± 50
Surface area of collecting ducts’ epithelial cells nucleuses (μm^2^)	65 ± 3	63 ± 3
NCO of collecting ducts’ epithelial cells	0.28 ± 0.02	0.26 ± 0.03
Number of connective tissue cells	141,133 ± 18,097	134,698 ± 12,922
Numerical density of connective tissue cells (mm^−3^)	22,990 ± 2366	22,133.4 ± 2982.2
Surface area of connective tissue cells (μm^2^)	99 ± 11	102 ± 6
Number of capillary endothelial cells	306,126 ± 24,065	296,854 ± 26,658
Numerical density of capillary endothelial cells (mm^−3^)	39,141 ± 3342	37,645 ± 4459
Surface area of capillary endothelial cells (μm^2^)	77 ± 4	75 ± 4
Surface area of glomeruli (μm^2^)	3086 ± 415	4060 ± 535 *
Bowman’s space	49 ± 4	44 ± 4

**Table 5:** Stereological parameters of the spleen of the control group and treated group; values are shown as mean ± SD (* *p* < 0.05; *t*-test).

Parameter	ALBO-OS	Control
Volume density of epithelial cells (mm^0^)	0.382 ± 0.036	0.359 ± 0.029
Volume density of lymphocytes (mm^0^)	0.409 ± 0.036	0.304 ± 0.058
Volume density of connective tissue (mm^0^)	0.119 ± 0.023	0.115 ± 0.025
Volume density of blood capillaries (mm^0^)	0.217 ± 0.04	0.167 ± 0.05
Number of epithelial cells	223,847.1 ± 27,129.8	204,568.3 ± 33,317.8
Numerical density of epithelial cells (mm^−3^)	37,498.5 ± 3318.7	35,641.8 ± 2625.9
Surface area of epithelial cells (μm^2^)	128.6 ± 2.7	131.5 ± 4.2
Surface area of epithelial cells’ nucleuses (μm^2^)	48.2 ± 5.3	41.2 ± 3.1
NCO of epithelial cells	0.274 ± 0.045	0.285 ± 0.052
Number of connective tissue cells	149,901.1 ± 6287.0 *	137,006.4 ± 8694.3
Numerical density of connective tissue cells (mm^−3^)	26,104.9 ± 2169.8	23,146.3 ± 2871.5
Surface area of connective tissue cells (μm^2^)	110.0 ± 4.6	105.6 ± 5.7
Number of capillary endothelial cells	241,697.6 ± 40,694.6	223,148.5 ± 20,204.4
Numerical density of capillary endothelial cells (mm^−3^)	39,905.4 ± 3588.8	34,969.4 ± 2599.3
Surface area of capillary endothelial cells (μm^2^)	78.6 ± 2.5	74.16 ± 2.3
Number of lymphocytes	432,527.8 ± 37,841.8 *	348,336.9 ± 23,324.7
Numerical density of lymphocytes (mm^−3^)	57,323.4 ± 2201.4 *	49,131.5 ± 3740.2
Surface area of lymphocytes (μm^2^)	97.2 ± 3.5	96.4 ± 4.9

**Figure A1:**
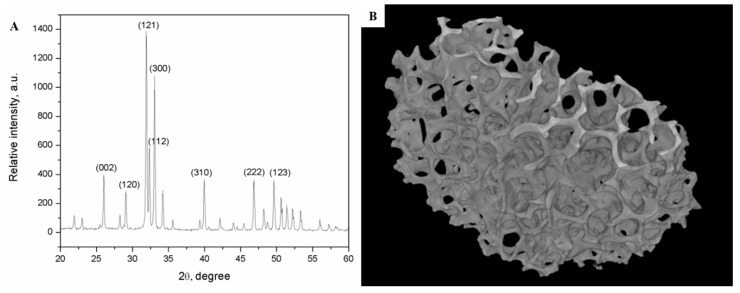
(**A**) XRD pattern of HA; (**B**) Micro-CT scan of nanoHAP-PLGA.

**Figure A2:**
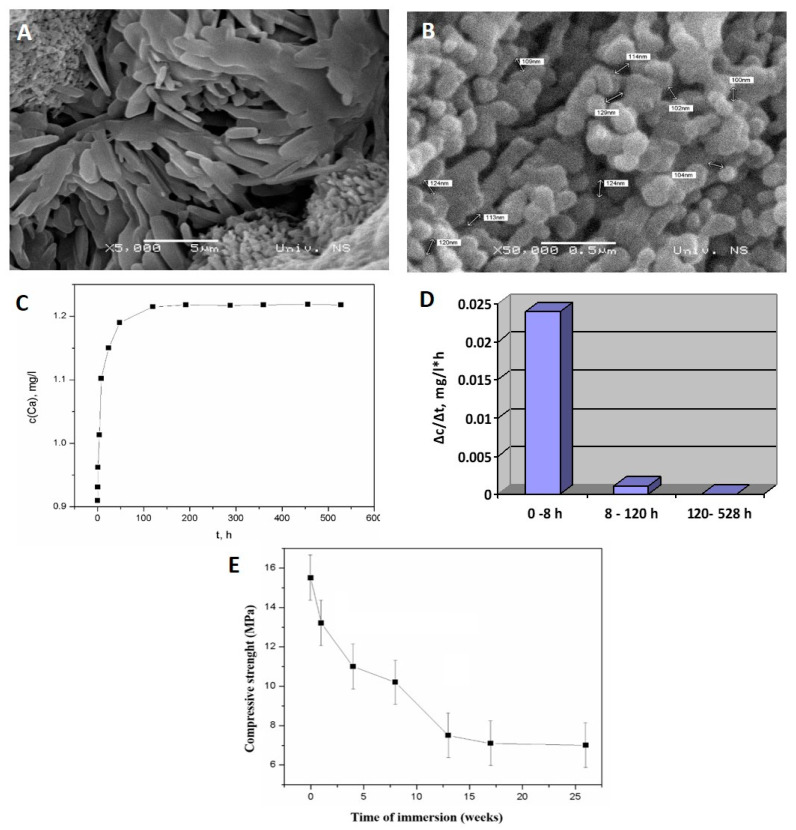
The SEM micrographs of nanoHAP-PLGA (**A**) 5000×, (**B**) 50,000×; (**C**) Change of the Ca^2+^ concentration in solution containing nanoHAP granules with time; (**D**) The rate of nanoHAP degradation during prolonged time period (0–428 h); and, (**E**) Change of compressive strength during nanoHAP degradation.

## Appendix A

### Appendix A.1. Physicochemical Characterization of the Extract of the ALBO-OS Scaffolds

X-ray diffraction (XRD) performed phase analysis of HA using a Philips PW 1050 diffractometer with Cu-Kα1–2 lamp. The data were collected in the 2*θ* range from 15–65°, in steps of 5°, and 2 s per step time exposure.

The microstructure of the ALBO-OS scaffold was investigated by micro-computed tomography (μCT) (SkyScan, Bruker 1172, Kontich, Belgium), and for the porosity analysis CTAn 1.14.4.1 software (SkyScan, Bruker) was used.

For the analysis of porosity, a classical method, known as the liquid displacement method, was also used. The absolute ethanol with density ρ was used as displacement liquid because of its ability to easily penetrate into the scaffolds without inducing the shrinkage or swelling of the PLGA. The samples, pycnometer, and ethanol were kept at 25 °C for 4 h prior to testing. A pycnometer that was filled with ethanol was weighed, W_1_. A scaffold sample of weight W_S_ was immersed into the bottle full of ethanol, and the bottle was then slowly surged until the air in the scaffold was all removed. At last, the pycnometer was refilled with ethanol and was weighed, W_2_. The scaffold saturated with ethanol was taken out of the pycnometer and the pycnometer was weighed, W_3_. The volume of the scaffold sample was calculated vs. = (W_1_ − W_2_ + W_S_)/ρ, as well as the total volume of the pores: Vp = (W_2_ − W_3_ + W_S_)/ρ. The porosity of the sample can be calculated by using equation: P = V_p_/(V_p_ + V_s_) = (W_2_ − W_3_ − W_S_)/(W_1_ − W_3_) [48].

Scanning electron microscopy (SEM) was used to analyze the material microstructure. For SEM observations samples were fixated in gradient ethanol concentrations (10–100%) and coated with gold by sputtering and JEOL JSM-5300 microscope was used.

In order to determine the rate of the decomposition/degradation of nanoHAP granules, they were submerged in mixture of the medium of a phosphate buffer (PBS) and Tris buffer (tris (hydroxymethyl) amino methane) in the ratio 1:1, with pH 7.37. The rate of degradation was determined as the difference in the calcium ions concentrations in the medium during experiment in comparison to starting concentration, and measured by inductively coupled plasma mass spectrometry (iCAP 6500 Duo ICP, Thermo Fisher Scientific, Inc. USA). For these purposes, aliquots of 3 mL of the medium were taken. The measurement started 10 minutes after immersion of the granules into the mixed medium and set according to the following schedule: 10 and 30 min., one, four, and eight hours, one, two, five, eight, 12, 15, 19, and 22 days. The obtained values are presented in the diagram depending on the calcium ions concentration.

Compressive strength of the granules ALBO-OS was measured after various immersion times in balanced salt Hanks solution (8 g NaCl, 0.35 g NaHCO_3_, 0.4 g KCl, 0.06 g KH_2_PO_4_, 0.1 g MgCl_2_ × 6H_2_O, 0.14 g CaCl_2_, 0.06 g Na_2_HPO_4_ × 2H_2_O, 0.06 g MgSO_4_ × 7H_2_O, 1 g glucose in 1000 mL of dH_2_O) with initial pH value 7.4. The samples were immersed into 10 mL of solution at 37 °C following the schedule. For the test of compressive strength, the porous compacts of nanoHAP were used, with completely equivalent structure of the ALBO-OS granules, with the dimension of 16 × 16 × 10 mm. For the measurement Instron model 4204 (Instron Corp., Canton, MA, USA) was used, with previous preparation of the samples by polishing with 600 and 1200 grit sandpaper on an automated rotary grinder, under gently running tap water, after which they were dried with adsorbent paper. The standard deviation was determined by measuring strength of the 10 samples.

### Appendix A.2. A Brief Review of ALBO-OS Structural and Physicochemical Characteristics

XRD revealed that the phase composition of the HA corresponds to carbonate calcium hydroxyapatite Ca_10_(PO_4_)_6_(OH)_2_ (JCPDS 9–432). All characteristic diffraction peaks were present: (002) at 25.98°, (120) at 29.03°, (121) at 31.87°, (300) at 33.07°, (310) at 39.94°, (222) at 2θ, and (123) at 49.54° (Figure A1A).

Very high porosity of ALBO-OS compact was revealed by liquid displacement method and particularly micro CT analysis (Figure A1B). The total porosity was 75.4%, which was also the value of the open porosity. Additionally, a significantly high number of connected pores per mm^3^ was found, 13.6 in value. Micro CT show very wide pore distribution, as shown in the Table A1.

**Table A1:** Values of ALBO-OS porosity.

Pore Sizes	Total Pore Volume (%)	Thickness Distribution of the Scaffold Inner Walls (%)	Number of Pores with Average Radius in the Given Range	Pores of Average Radius in the Given Range in Total Number of Pores (%)
0.06 mm–0.1 mm	2.52	8.90	9333	61.6
0.1 mm–0.2 mm	5.60	64.42	3294	21.7
0.2 mm–0.3 mm	5.83	26.68	737	4.8
0.3 mm–0.4 mm	11.66	/	971	6.4
0.4 mm–0.5 mm	17.44	/	379	2.5
0.5 mm–0.6 mm	18.18	/	216	1.4
0.6 mm–0.7 mm	16.96	/	119	0.8
0.7 mm–0.8 mm	15.06	/	72	0.5
More than 0.8 mm	6.75	/	25	0.2

From the Table A1, it is visible that although the average pore volume is the smallest for radius pore in the range 0.06 mm and 0.1 mm, its number is the highest (almost 2/3 total pore number). Most of them are in the radius range less than 0.02 mm, (83.3% of total pore number). From the other side, the number of pores with radius in the range higher than 0.4 mm is only about 5.4%.

The SEM micrographs clearly showed that the obtained scaffolds had very porous three-dimensional (3D) macrostructure (Figure A2). SEM examinations show a well-defined internal geometry, with fine pores of multimodal diameters, which is particularly visible in Figure A2B. The pores much higher than 5 μm and elongated grains sizes of 5–15 μm, can be noticed in the mean part of figure, while in the fragments given in the figure edges, very fine porosity can also be noticed, with elongated and spherical grains sizes order of the 120 nm in radius, being interconnected with surrounding grains, forming among them pores of similar radius (100 to 130 nm), and grains of mean diameters from 100 to 220 nm (clearly visible in the higher SEM magnification). The particles are mostly of polygonal shape, being mutually interconnected into clusters with pores in their centers and edges, forming characteristic micro- and nano-patterns.

#### Appendix A.2.1. Rate of ALBO-OS Degradation

Figure A2 provides the obtained results of the rate of degradation of the ALBO-OS. From the concentration of the calcium ions in saturated part od the solubility curve, the solubility product was determined. The obtained value of 7.85 · 10^−44^ was very close to the product solubility of biological apatite (3.51 · 10^−43^) (Figure A2C).

The rate of ALBO-OS degradation was examined during prolonged-time period (0–428 h), as it is shown in histogram in Figure A2D. Changes of the solubility rate as a change of concentration of calcium ions in the time unit show that in static system the rate of solubility is the extremely high for initial 8 h measured (0.024 mg/l·h), while, after 120 h, it significantly decreases (0.001 mg/l·h). It can be noticed that the peak of calcium ion release is present during first five days of immersion. Finally after 528 h it is almost neglectable (7.4 · 10^−6^ mg/l·h), similarly to the biological apatite.

#### Appendix A.2.2. Change of Compressive Strength during ALBO-OS Degradation

During six weeks of ALBO-OS immersion in Hanks solution, which has a composition close to the composition of body fluids, it can be noticed that compressive strength is changed from 15.5 MPa to 7 MPa. Further changes between 13 and 26 weeks were relatively low (Figure A2E).
